# Tissue-Specificity of Antibodies Raised Against TrkB and p75^NTR^ Receptors; Implications for Platelets as Models of Neurodegenerative Diseases

**DOI:** 10.3389/fimmu.2021.606861

**Published:** 2021-02-11

**Authors:** Samuel Fleury, Imane Boukhatem, Jessica Le Blanc, Mélanie Welman, Marie Lordkipanidzé

**Affiliations:** ^1^ Research Center, Montreal Heart Institute, Montreal, QC, Canada; ^2^ Faculty of Pharmacy, Université de Montréal, Montreal, QC, Canada

**Keywords:** platelet, neurotrophin receptors, tropomyosin receptor kinase B, brain-derived neurotrophic factor, pan-neurotrophic receptor p75^NTR^

## Abstract

Platelets and neurons share many similarities including comparable secretory granule types with homologous calcium-dependent secretory mechanisms as well as internalization, sequestration and secretion of many neurotransmitters. Thus, platelets present a high potential to be used as peripheral biomarkers to reflect neuronal pathologies. The brain-derived neurotrophic factor (BDNF) acts as a neuronal growth factor involved in learning and memory through the binding of two receptors, the tropomyosin receptor kinase B (TrkB) and the 75 kDa pan-neurotrophic receptor (p75^NTR^). In addition to its expression in the central nervous system, BDNF is found in much greater quantities in blood circulation, where it is largely stored within platelets. Levels 100- to 1,000-fold those of neurons make platelets the most important peripheral reservoir of BDNF. This led us to hypothesize that platelets would express canonical BDNF receptors, i.e., TrkB and p75^NTR^, and that the receptors on platelets would bear significant resemblance to the ones found in the brain. However, herein we report discrepancies regarding detection of these receptors using antibody-based assays, with antibodies displaying important tissue-specificity. The currently available antibodies raised against TrkB and p75^NTR^ should therefore be used with caution to study platelets as models for neurological disorders. Rigorous characterization of antibodies and bioassays appears critical to understand the interplay between platelet and neuronal biology of BDNF.

## Introduction

Platelets are circulating anucleate cells originating from megakaryocytes. In addition to their crucial role in hemostasis, platelets have been proffered as a peripheral model for the study of neuronal processes as they share many similarities with neurons (1–3). These include similar secretory granule types with homologous calcium-dependent secretory mechanisms (4–7) as well as internalization, sequestration and secretion of many neurotransmitters (8–10). Moreover, platelet abnormalities are reported in multiple neurological pathologies (11), suggesting common pathophysiological mechanisms. Indeed, platelets express many proteins found in neurons, including serotonin transporter SERT (12), amyloid precursor protein (APP), and amyloid β (2). The brain-derived neurotrophic factor (BDNF) is one such protein present in the central nervous system that is also found within platelets (13), with concentrations reaching up to 1,000-fold those of neurons (13–15).

In the brain, BDNF is involved in axonal growth through the binding of the tropomyosin receptor kinase B (TrkB). This receptor has a highly glycosylated extracellular domain (ECD) and an intracellular domain (ICD) consisting of a SRC homology 2 domain-containing-transforming protein C (Shc)-binding domain and a tyrosine kinase region (16, 17). Two truncated isoforms are also found in the central nervous system: the 95 kDa TrkB-T1 isoform, lacking both the Shc-binding and tyrosine kinase domains (18, 19) and the 100 kDa TrkB-T-Shc isoform that also lacks the tyrosine kinase domain but expresses the Shc-binding domain (19, 20). Additionally, BDNF is involved in the myelination of peripheral axons through the binding of the 75 kDa pan-neurotrophic receptor (p75^NTR^). This receptor is composed of an ECD consisting of four cysteine-rich domains (CRD) containing sites for both *N*- and *O*-linked glycosylation (21, 22). The intracellular domain consists of a palmitoylated chopper domain followed by a death domain (23, 24). The full-length isoform of the p75^NTR^ receptor has a monomeric molecular weight varying between 72 and 85 kDa (21, 25–30). A 62–65 kDa splice variant lacking CRD 2 through 4 has also been reported (27, 31).

Platelets internalize BDNF and secrete it upon activation (32, 33). While the contribution of the brain-borne BDNF to the platelet pool is still unclear, circulating levels of BDNF are associated with multiple neurological diseases, suggesting that peripheral BDNF could be used as a model of neuronal BDNF levels (34, 35). TrkB-T1 is found in megakaryocytes (36) and was recently reported in a platelet proteomic dataset (37); the presence of the p75^NTR^ mRNA has been reported in platelet transcriptomic studies (38–41). Nevertheless, BDNF receptors have not been reported using antibody-based approaches at the protein level (32, 42). To assess these divergences, we tested multiple antibodies against TrkB and p75^NTR^ receptors on platelets by immunoblotting and flow cytometry. Herein, we report important tissue-specificity among the multiple antibodies raised against TrkB and p75^NTR^ receptors, highlighting the importance of thorough antibody characterisation when investigating these receptors.

## Methods

### Antibodies and Reagents

Acid citrate dextrose solution A (ACD-A) was purchased from the Montreal Heart Institute pharmacy (DIN: 00788139). Prostaglandin E_1_ (PGE_1_, catalog no. 1620) was obtained from Tocris Bioscience. Antibodies against TrkB and p75^NTR^ are presented in **Table 1**. Allophycocyanin (APC)-Vio770 isotype control (catalog no. 130-104-618) was from Miltenyi Biotec. Mouse IgG_1_ (catalog no. MAB002) and IgG_2B_ (catalog no. MAB004) isotypes were from R&D Systems. Alexa Fluor 488-conjugated donkey anti-mouse IgG and donkey anti-rabbit IgG were from Thermo Fisher Scientific (catalog no. A21202 and A21206). The healthy human brain cerebral cortex full tissue lysate was obtained from Novus Biologicals (cat. NB820-59182). Proteins were extracted from the cortex of a healthy 66 years old male using the total protein extraction kit (cat. NBP2-37853). Proteins were aliquoted and conserved at −80°C until western blot analyses. U87-MG and U251-MG cells were a gift from Dr. Gaëlle V. Roullin. Eagle’s minimum essential medium (EMEM) and fetal bovine serum (FBS) were obtained from Wisent and Thermo Fisher Scientific. Deglycosylation kits were obtained from New England Biolabs (catalog no. P0704S and P6044). Platelet integrin Ibα (CD42b) and integrin αIIb (CD41) antibodies were from Santa Cruz Biotechnology (catalog no. sc-59051 and sc-365938). Platelet integrin β3 (CD61) antibody coupled to phycoerythrin (PE) and corresponding control isotype were from Miltenyi Biotec (catalog no. 130-110-749 and 130-104-613). Sortilin antibody was from Abcam (catalog no. ab16640) and lysosomal-associated membrane protein 1 (LAMP-1) was from the Developmental Studies Hybridoma Bank (catalog no. H4A3).

**Table 1:** List of antibodies tested.

Target	Manufacturer	Catalog number	Clone	Immunoblotting concentration	Flow cytometry concentration
TrkB	Abcam	ab134155	EPR1294	0.5 µg/ml in 5% skim milk	–
	Abnova Corporation	H00004915-M02	3D12	1 µg/ml in 3% BSA	–
	Alomone Labs	ANT-019	Polyclonal	1 µg/ml in 5% skim milk	–
	Biosensis	R-1834	Polyclonal	1 µg/ml in 3% BSA	–
	Millipore Sigma	HPA007637	Polyclonal	(1 µg/ml in 5% skim milk)	–
	Novus Biologicals	NBP2-52524	10B6C4	0.5 µg/ml in 3% BSA	–
	R&D Systems	AF1494	Polyclonal	1 µg/ml in 3% BSA	–
	R&D Systems	FAB397G * (FITC)	75133	–	80 to 330 µg/ml
	R&D Systems	FAB3971G (FITC)	72509	–	40 µg/ml
	R&D Systems	MAB397	75133	0.5 µg/ml in 3% BSA	(20 µg/ml)
	R&D Systems	MAB3971	72509	(0.5 µg/ml in 3% BSA)	20 µg/ml
	Sino Biologicals	10047-MM12	7H6E7B3	(1 µg/ml in 5% skim milk)	40 µg/ml
p75NTR	Alomone Labs	ANT-007	Polyclonal	0.8 µg/ml in 3% BSA	20 µg/ml
	Alomone Labs	ANT-011	Polyclonal	0.4 µg/ml in 3% BSA	–
	Biosensis	M-011-100	ME20.4	–	[20 to 100 µg/ml]
	EMD Millipore	05-446	ME20.4	–	80 to 200 µg/ml
	Millipore Sigma	HPA004765	Polyclonal	0.3 µg/ml in 5% skim milk	–
	Miltenyi Biotec	REA844 (APC-Vio770)	REA844	–	1:25
	Miltenyi Biotec	130-113-983 (PE)	ME20.4-1.H4	–	1:5
	Santa Cruz Biotechnology	sc-271708	B1	0.8 µg/ml in 5% skim milk	–

All other antibodies were indirectly labeled with either donkey anti-mouse or donkey anti-rabbit secondary antibodies conjugated to Alexa Fluor 488. Antibody concentrations for immunoblotting and flow cytometry are listed either in µg/ml, or in dilution factor of the stock concentration. *custom-made antibody, PE, phycoerythrin; APC, allophycocyanin;— antibody not used for this application; ( ), Antibody not validated for this application by the manufacturer, [ ], antibody supported but not validated for this application by the manufacturer.

For pre-conjugated antibodies, the associated fluorochrome appears in parenthesis next to the catalog number.

### Participant Selection

The study protocol was approved by the Montreal Heart Institute Scientific and Research Ethics Committee (#2018-2368) and written informed consent was obtained from each participant. Participants (four females and three males) were healthy adults aged between 22 and 43 years old, refrained from taking drugs known to affect platelet function in the 14 days before sampling, had not undergone major surgery in the last 6 months, did not have a history of bleeding symptoms and had platelet counts and hemoglobin levels within normal ranges.

### Blood Collection and Platelet Isolation

Whole blood was collected in syringes containing ACD-A anticoagulant (1:5 ratio) with 21G needles. Blood was centrifuged at 200g for 15 min and platelet-rich plasma (PRP) was collected. PGE_1_ (1 µM) was added to PRP to prevent platelet activation during isolation. PRP was centrifuged for 10 min at 1,000g to pellet platelets. The supernatant was discarded, and platelets were resuspended gently in Tyrode’s buffer (137 mM NaCl, 11.9 mM NaHCO_3_, 0.4 mM NaH_2_PO_4_•2H_2_O, 2.7 mM KCl, 5.6 mM glucose, 1.1 mM MgCl_2_, pH 7.4). The purity of the platelet preparation was verified by flow cytometry. The mean percentage of events contained within the platelet gate and verified by platelet integrin β3 (CD61) labeling was 99.53 ± 0.27%. A representative flow cytometry readout is shown as **Figure S1**.

### Cell Culture

U87-MG and U251-MG human glioblastoma cells were grown in EMEM supplemented with 10% FBS and 1% penicillin/streptomycin mix at 37°C and a fixed CO_2_ level of 5%. Cells were washed with phosphate-buffered saline (PBS) prior to trypsinization. Cells were then pooled down by 800g centrifugation and washed again in PBS prior to lysis for immunoblotting or fixation for flow cytometry experiments.

### Deglycosylation

Platelets or U87-MG cells were lysed in RIPA buffer (150 mM NaCl, 5 mM EDTA pH 8.0, 50 mM Tris-HCl pH 8.0, 1% NP40, 0.5% sodium deoxycholate, 0.1% SDS). Proteins from whole cell lysates were denatured in denaturing buffer (0.5% SDS, 40mM DTT, B1704S, New England Biolabs, MA, USA) and heated to 100°C for 10 min. Lysates were put on ice and glycobuffer 2, 1% NP40 and protein *N*-glycanase F (PNGase F) (P0704S, New England Biolabs, MA, USA) were added to denatured proteins for *N*-deglycosylation. Protein deglycosylation mix II (P6044, New England Biolabs, MA, USA) containing PNGase F, *O*-glycosidase, neuraminidase A, β1-4 galactosidase, and β-*N*-acetylhexosaminidase f was used for *N* and *O*-deglycosylation assays. Samples were incubated overnight at 37°C and then conserved at −80°C until analysis. Glycosylation profiles were assessed by mass shift on western blots, using the ANT-019 antibody for the TrkB receptor and the HPA004765 antibody for the p75^NTR^ receptor. Enzymatic activity was verified by reblotting membranes for *N-*glycosylated proteins sortilin and CD42b (*N*-glycosylation) or CD41 and LAMP-1 (*O* and *N*-glycosylation).

### Gel Electrophoresis and Immunoblotting

Platelets were centrifuged in the presence of PGE_1_ at 1,000g for 10 min, at room temperature (RT). The supernatant was discarded and platelets were lysed in ice-cold RIPA buffer containing protease and phosphatase inhibitors. 4X Laemmli’s buffer (250 mM Tris pH 6.8, 8% SDS, 40% glycerol, 20% β-mercaptoethanol, 0.02% bromophenol blue) was added 1:4 to samples and heated at 95°C for 5 min. Proteins were resolved on 8% polyacrylamide gels and transferred onto 0.45 µm PVDF membranes. Membranes were blocked in 3% BSA or 5% non-fat dry milk depending of the primary antibody diluent and incubated at 4°C overnight with the primary antibody. Membranes were then washed thrice for 10 min in Tris-buffered saline containing 0.1% Tween (TBS-T) and incubated in secondary antibody conjugated to horseradish peroxidase (Jackson ImmunoResearch Laboratories, West Grove, PA, USA) at a dilution of 1:10,000 in 5% milk for 60 min. Membranes were washed thrice in TBS-T and exposed to HRP substrate (Immobilon Classico Western HRP substrate, Luminata Classico, EMD Millipore, Etobicoke, ON, Canada). Chemiluminescence was captured on half-blue films (Mandel Scientific, Guelph, ON, Canada).

### Flow Cytometry

Platelets or U87-MG cells were fixed in 1% paraformaldehyde (PFA) at RT for 15 min. Following fixation, cells for intracellular labeling were permeabilized by adding 0.1% Triton X-100 for 10 min. Permeabilization was stopped by adding 500 µl of PBS. Cells were than pooled down and resuspended in PBS. Samples were labeled with primary antibody or with the corresponding isotypes for 30 min in the dark at RT. For unconjugated antibodies, secondary antibodies conjugated to Alexa Fluor 488 were added at a dilution 1:200 and incubated in the dark for 30 min. Cells were gated based on size and granularity. A total of 10,000 events were acquired with the MACSQuant Analyzer 10 flow cytometer; data was analyzed using the MACSQuantify software (version 8.2.1).

### Data Analysis

Immunoblotting data is representative of a minimum of three independent experiments and five healthy volunteers for platelets. For flow cytometry, data are presented as median (25^th^ percentile; 75^th^ percentile), corrected by isotype control and are representative of three or more independent experiments. Sample size varies between three for negative results (i.e., absent or very weak expression) and 10 for results suggestive of expression for increased precision.

## Results

### Antibodies Targeting TrkB

#### TrkB in the Brain and Platelets

**Figure 1:**
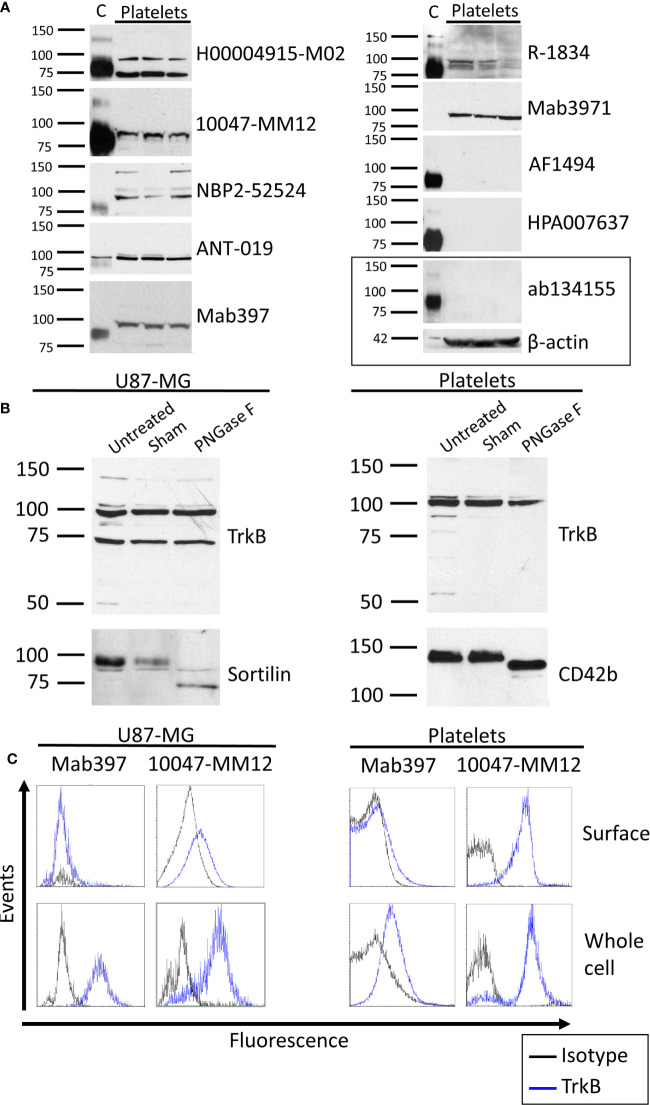
TrkB expression in human brain and platelets. **(A)** Healthy human cortex (C; 3 µg) and platelets lysates (platelets; 30 to 75 µg) were analyzed in denaturing and reducing conditions and blotted with antibodies raised against TrkB extracellular domain. Left: molecular weight marker in kDa. Right: antibody catalog number. β-actin was used as a loading control. **(B)** Human glioblastoma cells U87-MG and healthy platelets lysates were either left untreated (Untreated) or submitted to 37°C overnight in absence (Sham) or presence of *N*-deglycosylation enzyme PNGase F (PNGase F). Membranes were blotted with ANT-019 antibody against TrkB ECD. PNGase F activity was confirmed by reblotting membranes with antibodies against *N*-glycosylated proteins sortilin for U87-MG and CD42b for platelets. **(C)** Human glioblastoma U87-MG cells and healthy human platelets isolated from whole blood were fixed or fixed and permeabilized. Cells were labeled with antibodies directed toward the extracellular domain of the TrkB receptor and analyzed by flow cytometry. Results shown are representative of **(A)** ≥ 3 independent experiments and ≥ 5 different platelet samples from different donors and **(B, C)** 3 independent experiments and 3 different donors for platelets samples.

Except for mab3971, all antibodies tested identified the 95 kDa TrkB-T1 isoform in human cortex, while only half displayed the full-length isoform. Out of the 10 antibodies tested, seven showed a band directly under the 100 kDa weight marker in platelet samples (**Figure 1A** and **Figure S2**). This finding is in line with the observation of TrkB-T1 in primary human megakaryocytes by Labouyrie et al. (36). Furthermore, a band corresponding to the full-length TrkB receptor expected at a molecular weight of 145 kDa was displayed in platelets only by the NBP2-52524 antibody and was not present in platelets from all volunteers. As the full-length TrkB receptor has not yet been reported in platelets or megakaryocytes, further investigations to confirm the presence of this isoform in platelets is warranted. Interestingly, mab3971 displayed a band at the molecular weight of 95 kDa in platelet lysates but failed to detect the TrkB receptor in the human cortex. On the other hand, three antibodies identified at least the truncated isoform of the TrkB receptor in the cortex but failed to in platelet lysates. In addition, all six antibodies that identified the TrkB-T1 isoform in both cortex and platelets systematically displayed a lower band in the cortex, suggesting slight differences between TrkB-T1 in these samples. Interestingly, TrkB-T1 originating from brain tissues has also been observed to run at a slightly lower molecular mass than that of other cell types in mice, potentially due to differential glycosylation (18).

#### Deglycosylation of the TrkB Receptor

We then tested whether differences in TrkB mass could be explained by *N*-glycosylation. In both cell types, the protein identified by the TrkB antibodies was unaffected by PNGase F treatment (**Figure 1B**). Deglycosylation of glycoproteins sortilin in U87-MG and CD42b in platelets confirmed that PNGase F was active in the experimental settings. The absence of a mass shift following PNGase F treatment is in opposition to previous results reporting that TrkB is *N*-glycosylated (43), and argues against glycosylation as the primary cause of different molecular masses reported here and elsewhere (18).

#### Cellular Localization of the TrkB Receptor

Flow cytometry was used to assess the localization of the TrkB protein in human platelets. U87-MG cells were used as a positive control. A total of five antibodies were tested (**Figure 1C**, **Table 2**), including the mab397 and mab3971 in both direct (preconjugated to fluorochrome) and indirect labeling (conjugated with AlexaFluor488 secondary antibody).

**Table 2:** TrkB and p75^NTR^ expression assessed by flow cytometry.

Target	Catalog no./clone	Fluorochrome	Host	U87-MG cells		Platelets	
				Surface (%)	Whole cell (%)	Surface (%)	Whole cell (%)
TrkB	MAB397/75133	Alexa Fluor 488	Mouse IgG_2B_	2.6 (0.0; 14.3)	43.3 (26.8; 76.3)	7.7 (0.7; 60.2)	58.0 (34.4; 86.5)
	FAB397G*/75133	Pre-conjugated to FITC	Mouse IgG_2B_	0.1 (0.0; 1.2)	0.0 (0.0; 1.2)	0.0 (0.0; 0.0)	7.2 (0.0; 14.4)
	MAB3971/72509	Alexa Fluor 488	Mouse IgG_1_	0.0 (0.0; 0.0)	0.0 (0.0; 15.5)	3.3 (0.0; 13.8)	1.1 (0.4; 6.6)
	FAB3971G/72509	Pre-conjugated to FITC	Mouse IgG_1_	0.0 (0.0; 0.0)	0.0 (0.0; 0.0)	0.0 (0.0; 2.1)	0.0 (0.0; 18.9)
	10047-MM12/7H6E7B3	Alexa Fluor 488	Mouse IgG_1_	2.2 (0.0; 23.4)	39.1 (26.8; 69.9)	66.4 (10.6; 90.9)	79.7 (41.3; 97.2)
P75^NTR^	REA844/REA844	Pre-conjugated to APC-Vio770	Humanized IgG_1_	68.9 (43.2; 72.3)	77.3 (67.1; 87.8)	19.6 (7.6; 62.5)	56.4 (23.4; 71.6)
	130-113-983/ME20.4-1.H4	Pre-conjugated to PE	Mouse IgG_1κ_	16.9 (11.8; 23.7)	36.6 (21.4; 44.0)	16.2 (9.4; 27.8)	6.8 (5.1; 11.3)
	M-011-100/ME20.4	Alexa Fluor 488	Mouse IgG_1_	0.0 (0.0; 9.6)	0.0 (0.0; 3.0)	0.0 (0.0; 0.2)	0.0 (0.0; 0.0)
	ANT-007/polyclonal	Alexa Fluor 488	Rabbit polyclonal	0.0 (0.0; 12.7)	0.0 (0.0; 3.0)	0.4 (0.0; 21.8)	0.4 (0.0; 36.4)
	05-446	Alexa Fluor 488	Mouse IgG_1_	0.0 (0.0; 1.8)	0.0 (0.0; 15.7)	0.0 (0.0; 12.0)	33.2 (0.1; 82.7)

U87-MG cells and platelets were fixed in 1% paraformaldehyde for surface labeling and further permeabilized in 0.1% Triton X-100 for whole cell labeling. Cells were then labeled with antibodies raised against the extracellular domain of the TrkB or p75^NTR^ receptor. Data represent percentage of positive cells, corrected for isotype control, and are presented as median (25^th^; 75^th^ percentile). Data are representative of at least 3 independent experiments. FITC, fluorescein isothiocyanate; PE, phycoerythrin; APC, allophycocyanin.

All antibodies tested indicate either an absence or very weak TrkB expression at the membrane of U87-MG cells (**Table 2**). For permeabilized cell labeling, the 10047-MM12 antibody detected TrkB in approximately half of the cells, a result that was reproduced with mab397 through indirect labeling. However, the same clone failed to recognize TrkB when preconjugated to the fluorochrome. Similarly, mab3971 antibody showed a weak proportion of U87-MG cells expressing TrkB through indirect labeling and resulted in complete absence of this protein when preconjugated to FITC, thus failing to bind to the positive control.

In platelets, 10047-MM12 showed TrkB to be expressed at the surface of approx. half of the platelet population and this proportion increased to nearly 75% when platelets were permeabilized (**Table 2**). A similar pattern was seen with the indirectly conjugated mab397, albeit to a lesser extent; preconjugation with the fluorochrome abolished labeling, as for U87-MG cells. The other antibodies tested showed absence or very low expression of TrkB at the platelet membrane, and minimal increases in permeabilized platelets (**Table 2**).

In summary, only the unconjugated mab397 and 10047-MM12 showed convincing TrkB signals in U87-MG cells and both antibodies showed membrane and intracellular labeling of TrkB in platelets, with a stronger signal in the intracellular compartment, suggesting that TrkB is present both at the cell membrane and in the intracellular compartment.

### Antibodies Targeting p75^NTR^

#### p75^NTR^ in the Brain and Platelets

**Figure 2:**
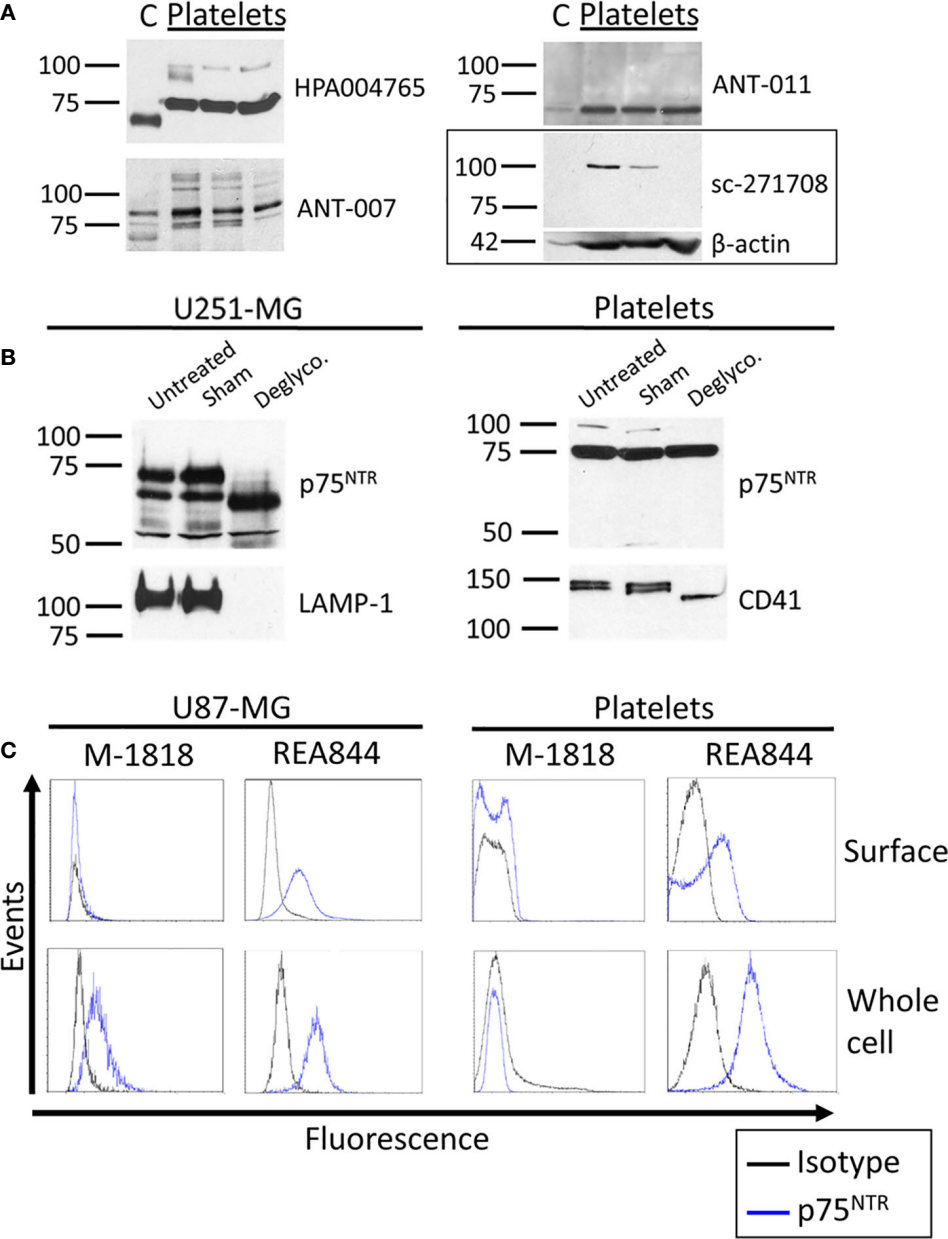
p75^NTR^ expression in human brain and platelets. **(A)** Healthy human cortex (C; 10 µg) and platelets lysates (platelets; 30 µg) were analyzed in denaturing and reducing conditions and blotted with antibodies raised against p75^NTR^. Left: molecular weight marker in kDa. Right: antibody catalog number. β-actin was used as a loading control. **(B)** U251-MG cells and healthy human platelet lysates were either left untreated (untreated) or submitted to 37°C overnight in absence (Sham) or presence of the protein deglycosylation mix II (Deglyco). Membranes were blotted with the HPA004765 antibody against p75^NTR^ ICD. Membranes were then stripped and reblotted for lysosomal-associated membrane protein 1 (LAMP-1) and CD41 as internal controls of enzymatic activity. **(C)** Human glioblastoma U87-MG cells and healthy human platelets isolated from whole blood were fixed or fixed and permeabilized. Cells were labeled with antibodies directed toward the p75^NTR^ receptor and analyzed by flow cytometry. Results displayed are representative of **(A)** ≥ 3 independent experiments and ≥ 5 different platelet samples from different donors and **(B, C)** 3 independent experiments and 3 different donors for platelets samples.

We then sought to investigate whether platelets expressed the low affinity BDNF receptor p75^NTR^ by western blotting (**Figure 2A** and **Figure S3**). The masses for the monomer of p75^NTR^ reported in the literature vary between 72 and 85 kDa, with 75 kDa being the most reported (21, 25–30). HPA004765 raised against the ICD and ANT-007 raised against the ECD both displayed bands at the expected size of 75 kDa corresponding to the monomeric form of the full-length receptor in platelet samples. ANT-007 also resulted in bands at around 80 kDa, and the ANT-011 antibody, which targets the ICD of p75^NTR^, resulted in a band at 70-72 kDa in platelets. sc-271708, also directed against the ICD, did not find any band in this range in platelets nor cortex lysates. Instead, it identified a protein running just above the 100 kDa marker that was only seen in platelet lysates. Interestingly, bands having the same mobility were also found with the HPA004765 and ANT-007 antibodies when used on platelets, but not on human cortex lysates. Furthermore, the intensity of this band in platelets is highly variable from an individual to another despite equal quantities of platelet lysates loaded into each well, as further supported by the β-actin used as a loading control. As with the TrkB receptor, the band found in the cortex lysates for p75^NTR^ runs slightly below that found in the platelet lysate samples, except for ANT-011 which identifies a single 72 kDa band both in both the human cortex and platelet lysates.

#### Deglycosylation of the p75^NTR^ Receptor

We investigated whether the differences in apparent molecular weights in the brain and platelet lysates originated from a differential glycosylation pattern. The p75^NTR^ receptor has a single *N*-glycosylation site on the first CRD of its ECD and multiple *O*-glycosylation sites on its stalk domain. We therefore used a deglycosylation mix that removes *N*-glycans as well as the majority of *O*-glycans. As shown in **Figure 2B**, the 75 kDa band identified in platelets did not shift following deglycosylation, while deglycosylation decreased the apparent molecular weight of this band in U251-MG cells. However, the higher band observed at approx. 100 kDa in platelets either disappeared or lost intensity in all 3 replicates. The fact that no new band appeared concomitantly to the loss of the 100 kDa band suggests that the mass shift engendered by deglycosylation caused the 100 kDa band to merge to the already present 75 kDa band, suggesting that this higher band could be a highly glycosylated form of the 75 kDa band observed in platelets.

#### Cellular Localization of the p75^NTR^ Receptor

We then used flow cytometry to assess p75^NTR^ localization in human platelets and U87-MG human glioblastoma cells used as a positive control. A total of five antibodies raised against the p75^NTR^ receptor ECD were tested, including ANT-007 also tested by western blotting, and the well-characterized clone ME20.4 (**Table 2**). The percentage of p75^NTR^-positive cells varied greatly depending on the antibody used, not only in platelet samples, but also in U87-MG cells (**Figure 2C** and **Table 2**). Only the humanized REA844 antibody and the clone ME20.4-1.H4 gave a positive expression signal in U87-MG cells. All the other antibodies tested showed close to no signal for both surface and whole cell labeling in U87-MG cells, including other ME20.4 clones. On platelets, antibodies REA844 and the clone ME20.4-1.H4 resulted in a signal at the cell surface. While the REA844 offered an increased signal in permeabilized platelets, clone ME20.4-1.H4 resulted in a weaker signal in permeabilized cells. In summary, only the REA844 antibody showed convincing signals in both U87-MG cells and platelets, with large disparities between antibodies in their ability to bind the receptor in U87-MG cells contributing to the uncertainty of the results seen in platelets.

## Discussion

We set out to identify TrkB and p75^NTR^ on human platelets using antibody-based techniques and tested various commercial antibodies from different host species and targeting different epitopes. While both receptors could be detected on human platelets, we found major discrepancies among antibodies in their ability to detect BDNF receptors on platelets, but also on human cortex and U87-MG cells. These results highlight important challenges in using antibody-based assays to determine the expression pattern of these receptors, with a notable lack of reproducibility among the tested antibodies.

### TrkB on Platelets

Our immunoblotting experiments suggest the presence of a truncated form of the TrkB receptor in human platelets (**Table 3**). However, major discrepancies were found among antibodies, as well as between brain and platelet lysates. All antibodies that worked on both samples consistently identified TrkB in the cortex at a slightly lower mass to that seen in platelets. A similar band shift was also reported for TrkB-T1 in mice, with brain lysates running slightly below NIH/3T3 cells (18). The authors suggested the mass shift was likely due to differential glycosylation but did not confirm their hypothesis. A similar hypothesis was also raised by another group facing challenges to identify TrkB in glial cells (44). Glycosylation on TrkB appears to be important for antibody recognition, as Eager et al. showed that the glycans on the ECD of TrkB were necessary for antibody binding, either by being included in the epitope or by allowing the correct epitope conformation for antibody binding (45). However, PNGase F treatment did not alter the apparent molecular weight of the bands detected by TrkB antibodies in platelets nor in the U87-MG cell line in our study. Because the intensity of the observed 95 kDa band did not decrease following PNGase F treatment, it seems unlikely that this is the result of a lack of affinity of the tested antibodies for the deglycosylated TrkB receptor. A limitation regarding these experiments is that while PNGase F has a large spectrum, it does not cleave all *N-*linked glycans. In addition, we focused on the glycosylation profile but cannot exclude that the observed mass difference seen by western blotting could be due to other post-translational modifications.

**Table 3:** Summary of antibody performance for TrkB.

TrkB						
Manufacturer	Catalog no.	Host	Detection in cortex		Detection in platelets	
			TrkB-FL	TrkB-T1	TrkB-FL	TrkB-T1
Abnova Corporation	H00004915-M02	Mouse	+	+	–	+
Sino Biologicals	10047-MM12		+	+	–	+
Novus Biologicals	NBP2-52524		–	+	+	+
R&D Systems	Mab397		–	+	–	+
R&D Systems	Mab3971		–	–	–	+
Alomone Labs	ANT-019	Rabbit	–	+	–	+
Biosensis	R-1834		+	+	–	+
Millipore Sigma	HPA007637		+	+	–	–
Abcam	ab134155		+	+	–	
R&D Systems	AF1494	Goat	–	+	–	–

This table summarizes the results obtained regarding the detection of the full-length (FL) and truncated (T1) isoforms of TrkB. +, detected; -, not detected.

It has been suggested that different TrkB glycosylation patterns could result in an alternative folding of the protein and alter the layout of certain epitopes (44, 45). This could explain the many differences between antibodies observed in flow cytometry, as this technique labels proteins in their native conformation, rather than under reducing conditions that can be used in immunoblotting. Nonetheless, it cannot explain the differences observed between the same clones through direct and indirect labeling. As we adjusted for non-specific binding by subtracting the isotype control fluorescence in our experiments, the difference is unlikely to arise from non-specific binding. Whether steric hindrance of fluorochrome-conjugated antibodies could explain the lack of binding would merit further attention with alternative fluorochrome conjugates. Taken together, these results highlight the importance of confirming findings with independent antibodies and characterizing them against known controls.

### p75^NTR^ on Platelets

Our immunoblotting experiments suggest the presence of the p75^NTR^ receptor in human platelets (**Table 4**). The 72, 75, and 80 kDa bands identified in platelets by the HPA004765, ANT-007 and ANT-011 antibodies all correspond to molecular weights reported for the full-length p75^NTR^ receptor (21, 25, 26, 28–30). Previous studies found that the apparent mass of the receptor varied depending on reducing conditions (25, 30). While the masses reported herein are in the same range, all samples were subjected to identical reducing conditions. Thus, the differences in weights cannot be attributed to variable reducing conditions. Furthermore, HPA004765 and ANT-007 antibodies identified the isoform in the cortex to run slightly below the isoform found in platelets. Despite the many glycosylation sites, *O* and *N*-deglycosylation did not lower the apparent molecular weight of the protein in platelets, in contrast with U251-MG cells as previously reported (25, 29). Whether other post-translational modifications, such as palmitoylation, may be the cause of the mass differences observed (46), and could explain the important discrepancies observed among the tested antibodies in flow cytometry, have not been the center of investigation so far.

**Table 4:** Summary of antibody performance for p75^NTR^.

p75^NTR^					
Manufacturer	Catalog no.	Host	Epitope	Detection in cortex	Detection in platelets
Santa Cruz Biotechnology	sc-271708	Mouse	ICD	–	?
Millipore Sigma	HPA004765	Rabbit	ICD	+	+
Alomone Labs	ANT-007		ECD	+	+
Alomone Labs	ANT-011		ICD	+	+

This table summarizes the results obtained regarding the detection of the full-length p75^NTR^ receptor. +, detected; −, not detected; ?, uncertain. ECD, extracellular domain; ICD, intracellular domain.

While bands between 72 and 85 kDa have all been associated to the monomeric p75^NTR^ receptor (21, 25–30), the band at 100 kDa is rather associated to a single p75^NTR^ receptor bound by dimeric nerve growth factor (NGF) (47, 48). However, the study of such a complex requires cross-linking, which we have not carried out, and the denaturing and reducing conditions used in our experiments render the possibility of a non-covalent complex unlikely. The intensity of the 100 kDa band was decreased or completely abolished by deglycosylation, suggesting either a highly post-translationally modified form of the receptor or a strong complex stabilized by glycans. The fact that this band is displayed by three antibodies targeting different epitopes suggest that this band is specific, and the nature of the protein or protein complex it identifies in platelets warrants further investigation.

### Platelets as Neuronal Biomarkers for BDNF Receptors

Several studies have highlighted alterations in brain TrkB and p75^NTR^ receptors in neurological disorders. For instance, the TrkB receptor levels have been shown to be decreased in the brain of schizophrenic patients (49), while alterations in p75^NTR^ cleavage is believed to lead to neuronal death in Alzheimer’s disease through the production and binding of amyloid β (50). The identification of the TrkB and p75^NTR^ receptors in platelets opens new avenues of research, using platelets as peripheral biomarkers of neurological expression patterns of these receptors.

Whereas circulating BDNF levels have been extensively studied (34), little is known about the interplay between BDNF, proBDNF, TrkB, and p75^NTR^ in these easily available peripheral cells (51, 52). The results presented herein highlighting structural differences between TrkB and p75^NTR^ receptors in human cortex and platelets, raise the possibility that these receptors in platelets might not be a true reflection of expression in the cerebral cortex. A better characterization of the activity of TrkB and p75^NTR^ in platelets is warranted, to assess whether BDNF receptors in platelets have an inherent biological role, and could potentially be used to mirror receptor function in neuronal tissues.

## Limitations

While the many antibodies characterized represent a strength of the present study, there are also noteworthy limitations. The fact that all antibodies were tested on the exact same cortex sample allowed a better comparison between antibodies themselves because the differences observed for the cortex sample were not due to unequal levels of receptors in the sample. However, we recognize that the cerebral cortex might not be representative of other brain regions, and the ratio between the multiple isoforms of BDNF receptors are known to vary from a region to another (53). Moreover, using a single donor does not allow representation of inter-individual variation in levels of these receptors in cortical tissues. The presence of TrkB and p75^NTR^ has been shown solely with antibody-based techniques. Since platelets are anucleate cells, we could not verify antibody specificity in this cell type using classic molecular biology approaches, such as knockout models, and have had to rely on cross-verification with independent antibodies, in the presence of cells/tissues with confirmed TrkB and p75^NTR^ expression. It is important to reconcile the results presented herein with reports of absence of TrkB or p75^NTR^ receptors on human platelets (32, 42). The fact that only the truncated isoform of TrkB lacking its tyrosine kinase domain has been found in platelets in our study may explain why prior reports using antibodies targeting the intracellular domain of TrkB receptors would have failed, highlighting the importance of confirming results with independent antibodies targeting different epitopes of the receptor. Arguably, protein sequencing by techniques such as mass spectrometry would further increase confidence in the results.

## Conclusion

Our results suggest that human platelets express a truncated form of the TrkB receptor and the full-length p75^NTR^ receptor; structural or post-translational differences from the isoforms expressed in the central nervous system are apparent on receptor mass. An important aspect of this work is the tissue-specificity of some antibodies targeting BDNF receptors, and a lack of reproducibility between antibodies, even within the same clonal selection. This highlights the importance of careful characterization of antibodies when using immuno-based assays to study BDNF receptors, both within and beyond the central nervous system. A thorough characterization of the TrkB and p75^NTR^ isoforms in platelets and other circulating cells is therefore critical before they can be recommended as models of neurocognitive health.

## Data Availability Statement

The raw data supporting the conclusions of this article will be made available by the authors, without undue reservation.

## Ethics Statement

The studies involving human participants were reviewed and approved by Montreal Heart Institute Scientific and Research Ethics Committee. The participants provided their written informed consent to participate in this study.

## Author Contributions

SF has performed assays, collected data, analyzed and interpreted data, and wrote the manuscript. IB, JLB, and MW have performed assays, collected data, analyzed and interpreted data, and critically revised the manuscript. ML has overseen the research group, assured funding, designed the research, analyzed and interpreted data, and critically revised the manuscript. All authors contributed to the article and approved the submitted version.

## Funding

This work was supported by the Canadian Institute of Health Research (PJT-159569), the Canada Foundation for Innovation Leaders Opportunity Fund (32797), and by trainee scholarships from the Faculté de pharmacie de l’Université de Montréal (SF, IB, and JLB), from the Faculté des études supérieures et postdoctorales of the Université de Montréal (SF and IB) and from the Canadian Vascular Network (SF). ML was supported by the Fonds de recherche du Québec en Santé (FRQS) Junior 1 Research Scholarship (33048); and is a Canada Research Chair in Platelets as biomarkers and vectors (950-232706).

## Conflict of Interest

ML has received speaker fees from Bayer; has participated in industry-funded trials from Idorsia; has served on advisory boards for Servier; and has received in-kind and financial support for investigator-initiated grants from Leo Pharma, Roche Diagnostics, Aggredyne, and Fujimori Kogyo.

The remaining authors declare that the research was conducted in the absence of any commercial or financial relationships that could be construed as a potential conflict of interest.
