# Supplementary material for: Tissue-Specificity of Antibodies Raised Against TrkB and p75NTR Receptors; Implications for Platelets as Models of Neurodegenerative Diseases
Source: Front Immunol. 2021 Feb 11;12:606861. doi: 10.3389/fimmu.2021.606861 (PMC7905091; doi:10.3389/fimmu.2021.606861)
Supplement: Supplementary file 1 [file DataSheet_1.pdf]

Supplemental material to:

Tissue-specificity of antibodies raised against TrkB and $p75^{NTR}$ receptors; implications for platelets as models of neurodegenerative diseases

Samuel Fleury[1,2,#], Imane Boukhatem[1,2,#], Jessica Le Blanc[1,2], Mélanie Welman[2], Marie Lordkipanidzé[1,2,*]

[1] Faculty of Pharmacy, Université de Montréal, Montréal, Québec, Canada;
[2] Research Center, Montreal Heart Institute, Montréal, Québec, Canada;
[#] These authors contributed equally to this work and are considered first authors

*** Correspondence:** Marie Lordkipanidzé
marie.lordkipanidze@umontreal.ca

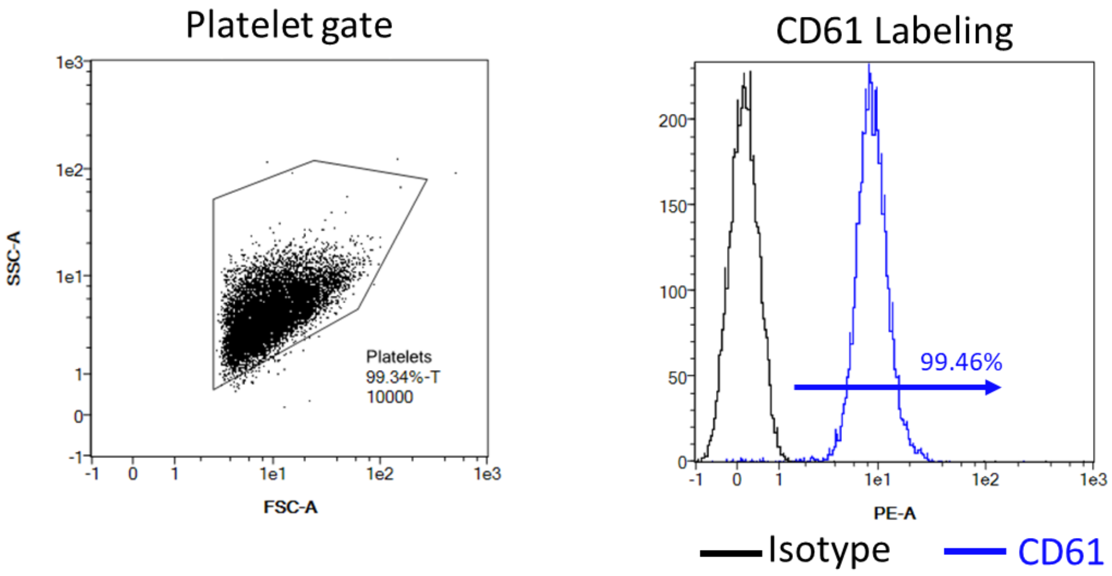

Figure S1: Purity of washed platelet preparations. Washed platelets were isolated by differential centrifugation as described in the methods section and the purity was assessed by flow cytometry. On the left: platelet gate based on size (forward scatter; FSC) and granularity (side scatter; SSC). On the right: fluorescence of events contained within the platelet gate when labeled with platelet integrin β3 (CD61; blue) or control isotype (black). More than 99% of the events within the platelet gate were CD61 positive, confirming that these events are indeed platelets.

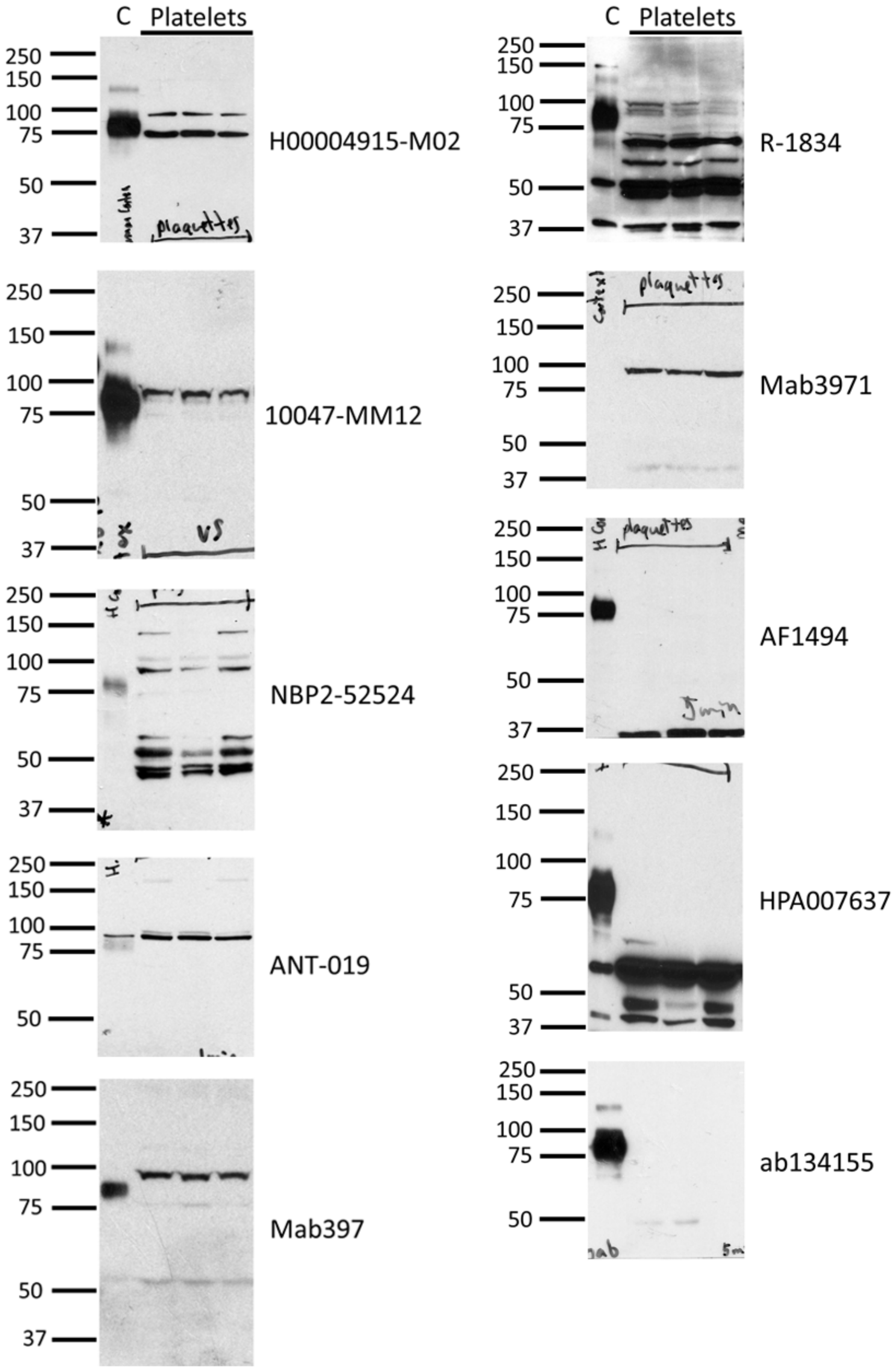

Figure S2: Complete TrkB western blots. Complete blots are shown for each of the 10 antibodies tested against the extracellular domain of the tropomyosin receptor kinase B (TrkB) receptor. C: Healthy human cerebral cortex full tissue lysate. Each blot is representative of 3 independent experiments on 5 or more healthy blood donors.

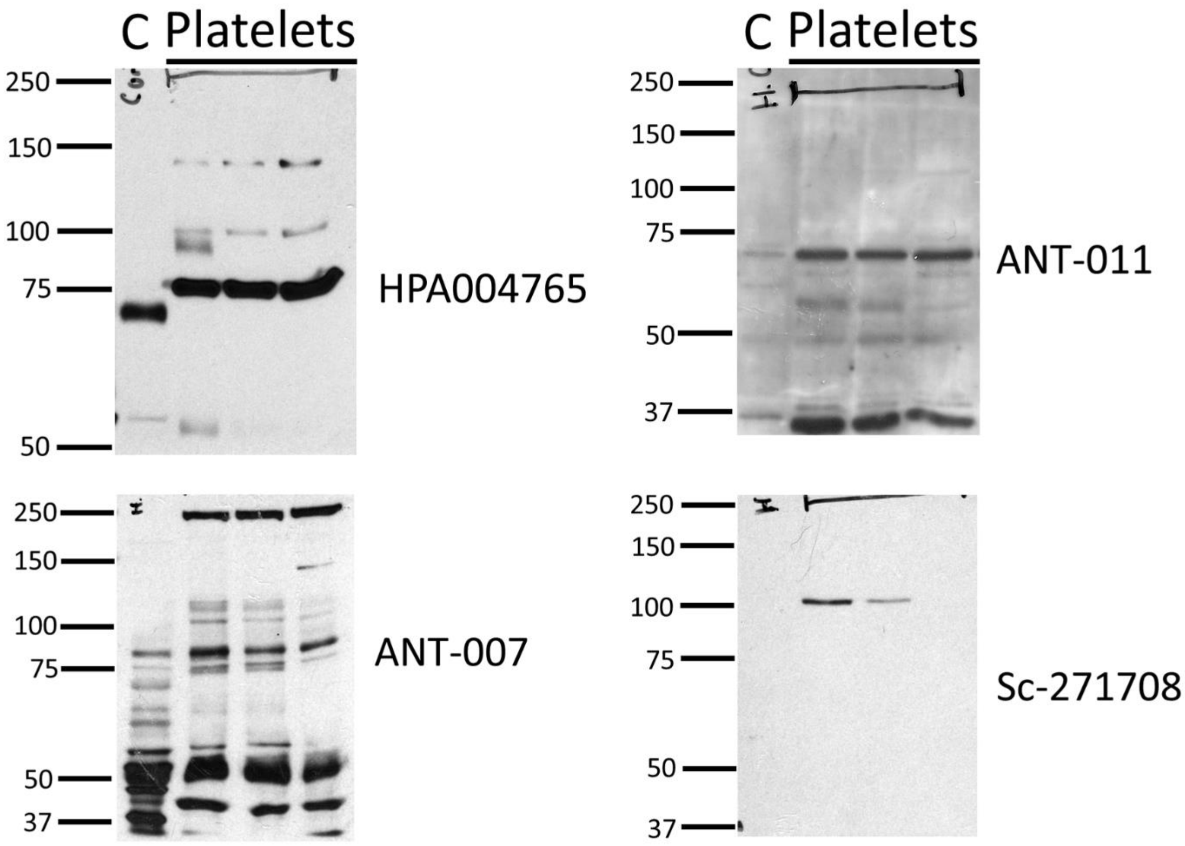

Figure S3: Complete p75^{NTR} western blots. Complete blots are shown for each of the 4 antibodies tested against the 75 kDa pan-neurotrophic receptor (p75^{NTR}). C: Healthy human cerebral cortex full tissue lysate. Each blot is representative of 3 independent experiments on 5 or more healthy blood donors.
